# Which Method of Root-Canal Filling Will Completely Obliterate Space?

**Published:** 1895-09

**Authors:** Safford G. Perry

**Affiliations:** New York


THE
### International Dental Journal.

Vol. XVI. September, 1895. No. 9.

### Original Communications.[1]

### WHICH METHOD OF ROOT-CANAL FILLING WILL COMPLETELY OBLITERATE SPACE?[2]

BY SAFFORD G. PERRY, D.D.S., NEW YORK.

In the half of the evening allotted to me there is not time for a full consideration of the different methods employed in filling the root-canals of the teeth, and I may as well proceed at once to describe the one that I believe to be the best, and the one I have adopted after many years' close study of the whole subject.

Before I can do that intelligently, however, it will be necessary to consider at some length the matter of the approach to the roots that are to be cleaned and filled. The methods I employ for the most part require openings to be made through the teeth directly over the roots in a line with their long axis, so that their canals can be entered with nearly straight instruments. The methods of cleaning and filling the canals, which will be described later, will show this to be an almost indispensable condition. If the cavities of decay do not allow this fairly direct approach, then tap-holes are to be drilled through the tooth, so that the roots can be entered in a nearly straight line. Leaving all cavities out of the question,

---

[1] The editor and publishers are not responsible for the views of authors of papers published in this department, nor for any claim to novelty, or otherwise, that may be made by them. No papers will be received for this department that have appeared in any other journal published in the country.

[2] Read before the New York Odontological Society, April 16, 1895.

and first considering sound teeth that are dead and have to be opened, we have then only to regard the fissures in the crowns, and to remember the anatomy of the roots, in order to know exactly where to drill. No mention need be made of the superior and inferior incisors, further than to say that, as a matter of course, they are to be drilled on their lingual sides, and at such angles as will give access to their canals in nearly a straight line. The first inferior bicuspids must be drilled through the small anterior crown fissure; the second bicuspid through the anterior division of its single irregular fissure. The first lower molar, not through the deep fissure near the middle of the crown, but just anteriorly to it, so that the tap-hole will come nearly over the anterior root. The tap-hole should then be reamed posteriorly to give access to the posterior root. The backward slant of this root is such that nothing more is needed to give direct access to its canal.

Then, substituting a right-angle hand-piece, the same tap-hole should be reamed towards the buccal and the lingual sides of the tooth, and this will open up the two canals in the anterior root, so that nearly straight instruments can be passed to their extremities. The inferior second molar should be entered just anteriorly of the deep middle fissure, so that the anterior root can be entered in nearly a straight line. A little reaming posteriorly will give access to the posterior root, as in the case of the first molar. The inferior wisdom tooth should be entered, also, just a trifle in front of the centre of the crown. The superior first bicuspid should be entered through the anterior depression of the fissure. This will give direct access to the two roots, if they exist. The second superior bicuspid, of course, through the fissure in the centre of the crown. The superior first molar should be entered through the anterior deep fissure, and the tap-hole reamed towards the palatine and buccal roots. The superior second molar should be entered through the anterior fissure in the same way. The superior wisdom-tooth should be entered from the anterior edge of its irregular fissure. The sizes of these tap-holes must vary with the different teeth. They must be as small as possible, in order not to weaken the teeth; but, on the other hand, they must be large enough to allow the entrance of light, and to give a chance for accurate work.

One naturally shrinks from drilling a hole in a sound tooth, but it is a necessary means to an end, and the patient had better have a weakened tooth with healthful roots than diseased roots and a sound, strong tooth.

Thus far I have assumed the absence of cavities. If they occur in the centre of the crowns, or on the anterior approximal surfaces of the molars and bicuspids of either jaw, tap-holes may not be necessary; but if they occur on the posterior approximal surfaces of these teeth, and are not very large, tap-holes are indispensable. If they are not considered so for the use of the instruments generally employed for cleaning and filling the roots, they certainly are for the use of the instruments I shall describe. A large cavity in the posterior surface of a lower molar may give direct access to the posterior root. In that event the tap-hole can be drilled more directly over the anterior roots. In such cases I have many times drilled two small holes over the front root, one over each canal, throwing the light by reflection through the cavity of decay. In the same manner a large cavity on the posterior surface of the superior molar may give direct access to the palatine root, and two tap-holes, one over each of the buccal roots, will give direct access to their canals. Often the tooth substance between these two tap-holes can be cut away with a fissure-bur, leaving a slot through which work is facilitated, and without much weakening of the tooth. This slot will be nearly half-way between the bottom of the deep fissure and the tips of the buccal cusps. In this case, also, light by reflection can be afforded through the cavity of decay, and the tap-holes can be very small and the slot very narrow.

If these large cavities on the posterior surfaces of molars do not quite give direct access to the posterior or palatine roots, a fissure-bur readily cuts a groove in the grinding-surface edge of the cavity, and a tapered root-canal reamer, revolved on the posterior side of the opening into the canals, opens up the root so that a straight broach wrapped with cotton can be rotated in it, and this rotation may be essential to its thorough cleansing. The reamers used for this purpose are those I presented before this Society several years ago. They are never used, except for opening the orifices of root-canals. They have a very gradual taper, so that no shoulder or irregularity of surface is left, as often occurs with the reamers generally in use. Cutting, as they do, on their sides, they are very dangerous instruments to use deep in the roots, and their use is therefore restricted to opening the orifices of the canals. And here let me say that I never use any of the means employed for enlarging the canals, except at their orifices, as described.

The roots of teeth are often very crooked, and some are very flat and thin, and an immense number of valuable teeth have been lost by heroic drilling through their sides. An untouched canal is always smooth, and, though curved, its extremities can almost invariably be reached with the proper instruments.

I will not say that I never use a fine barbed broach for the removal of freshly-devitalized pulps, but I will say that I seldom use them, even for that purpose, and I do not use them in any canal in which a pulp has been for some time dead. In their place I use the well-known Swiss broaches, made for watch-makers, and which can be found at any of the places where jewellers get their supplies. They are four-sided, and highly polished and spring-tempered. I place a dozen or two of them in a glass tube, and draw the temper to a deep blue, over an alcohol lamp or a Bunsen burner. The glass protects them from currents of cold air, allows them to cool slowly, and enables one to see the color of the steel. On these tempered instruments shreds of cotton or raw silk can be lightly or loosely rolled, so that it can be withdrawn from the root of a tooth, or left in it, as desired. If tightly rolled, it can be entangled in a devitalized pulp in such a way that the whole pulp can be removed with considerable precision. The silk or cotton, though very tightly rolled, is yet easily removed from the instrument by laying it between the folds of a napkin and pulling it off with the thumb and finger. If it has been used in a root long dead, the fingers can be protected from the bad odor of the cotton or silk by the use of a piece of rubber dam placed outside of the napkin.

There are many advantages in the use of this instrument in all the root-canal operations. In the first place, the fact that the broach is square makes it possible to roll the silk or cotton upon it with any degree of looseness or firmness in an instant of time. This, of course, cannot be done with a round, smooth broach. Having no barbs, the cotton or silk is equally quickly removed between the thumb and finger. Also, having no barbs, there are no weak places along its whole length, and it does not readily break. Every barbed instrument is weakened at the base of every barb; even a cross-scratch on any round instrument determines the place where it will break, if it break at all. If the barbed instruments do not break off entirely, the barbs almost invariably do, and in the canals, from which they cannot be removed. Besides, the barbs increase the diameter of the instrument, and it is no wonder that it is generally accepted that the fine canals of the anterior root of the first lower molar and the buccal roots of the superior molars cannot be entered to their extremities.

Swiss broaches can be procured that are almost hair-like in size, and their diameter is but slightly increased by a few fibres of silk or cotton, and they can be carried almost invariably to the end of every root-canal.

Charged with carbolic acid and used with patience, the living fibrils of pulp in a minute canal can be cauterized and destroyed, and in a blackened and disintegrated form can be removed little by little. It takes time, but finally the end of the root will be reached, and after the use of a great many rolls of silk it will finally come out of the canal, white and dry and clean. Or, if the root has been long dead, the same persistent effort will bring it out clean and comparatively odorless. By rotation of the instrument the contents of the canal become entangled in the silk and are removed. This rotation of the instrument is very essential, and now it can be seen why it is so necessary to open the teeth in such a way that a straight instrument can be used. These slender instruments can be employed in a wonderful way if they are kept straight, but they become unmanageable if they are bent.

For places where a stiff instrument is needed, I have points made from untempered piano-wire, but filed square, so that the cotton can be rolled on them in the same ready way. They are tapered like a trout-rod. They are stiff near the shank, and are therefore very manageable, and can be bent to enter a canal where direct access to it cannot be easily had.

The method by which these points are fastened to the handles originated with Dr. Darby. They are soldered to a little shank, and that is threaded and screwed into the socket-handle. In this way they are held firmly. This is a little thing, but it has been of great value to me.

For holding the Swiss broaches I have little handles turned from rosewood. They are tapered to a small diameter at the end, held by the thumb and finger. This is to facilitate rolling the cotton on the instrument. Of course, the smaller the diameter the faster it rolls. In the end that holds the instrument a hole is drilled, and the instrument, with a few fibres of cotton rolled on it, is easily fitted, and easily removed if it becomes broken. Into the end of the large handles that hold the stiff instruments a little wire is driven, which, held between the thumb and finger, enables one to rotate the instrument rapidly in rolling the silk or cotton on the point.

I have taken all this time to describe these instruments minutely because their use leads up to, and gives the key to the method I employ in filling the roots.

There may be nothing new to say on the subject of filling root-canals of good size. The posterior roots of lower molars, the palatine roots of superior molars, and the roots of the bicuspids and of the incisors, are probably filled by most operators either with chloro-percha, followed with the gutta-percha points, or with oxychloride, followed by the same points, in a manner that may be proof against the most searching criticism. These gutta-percha points originated in my office, and I have watched the headway they have made, naturally, perhaps, with considerable interest. They were suggested to me years ago by the use of the delicate paper points for drying the canals, described before this Society in a paper called "Offerings," by Dr. J. F. P. Hodson.

I think it will be generally admitted that the anterior roots of lower molars, and the buccal roots of superior molars, and very minute canals in general, have not only not been accurately filled, but have not always even had their pulps completely destroyed and removed, and in old, dead teeth they have not always been cleaned. I will not go so far as to say that with the delicate Swiss broaches, in the way described, it is possible to get to the end of all these inaccessible canals, but I will state that it is a practical thing to do so in almost every case. It is not only possible to get the canals clean, but it is also possible to fill them with a considerable degree of accuracy. If the hair-like broach, which is used for cleaning one of these fine canals, were wrapped with silk or cotton, and that charged with oxychloride or with chloro-percha, and then carried down to the closed end of the root (and these fine canals are always practically closed), and it could be left there, no one could deny that the canal must be filled with all the accuracy that could be expected in any root-filling. But, of course, the steel wire would not be permissible. When it is removed a space is left. Some of the chloro-percha or oxychloride may remain. We may repeat this process, and still there will be the same result.

Let us not deceive ourselves by believing that into such a minute hole, closed at one end and full of air, we can pump either of the materials named in such a way as to obliterate all space. With a minute opening at the end of the root for the escape of the air, it might be done, but in the roots of teeth in the mouth it is an impossibility. If we wrap the fibres of silk or cotton loosely so that they slip off the instrument and remain, still there is the space left by the instrument when it is removed. The only way is to leave the instrument itself. This result I accomplish by substituting gold or platinum wire, in the following manner:

The end of a piece of gold wire, smaller than the diameter of the canal to be filled, is bent over on itself with the pliers, and crimped to a uniform size in a fine groove in the beaks of the pliers. The length of the portion bent over is not more than the diameter of an ordinary pin. This is done in order to get a better hold of the wire if it ever needs to be removed from the root. In attempting to pull it out, if it does not come, but straightens out, there is then wire enough to get hold of with suitably made pliers. This doubled end of the wire is then fastened in a delicate pin-vice made for the purpose.

The depth of the canal having been measured by slipping on the Swiss broach one of the little rubber disks cut from the rubber dam by the Ainsworth punch, the wire is cut to the right length, and then, being firmly held by the pin-vice, is filed square to a tapering point by a very fine file. On this wire a half-dozen or a dozen fibres of silk or cotton are rolled exactly as is done on the Swiss broach. This is then transferred from the pin-vice to a plugger-point, into the end of which a hole is drilled, large enough to receive the doubled end of the gold wire. The fit is close enough to hold, and yet so loose that, when the gold wire has been put to its place in the root, the plugger slips off and leaves it in place.

This gold wire, while held in the plugger-point, is dipped in chloro percha or oxychloride of zinc and put at once to its place in the root, the canal having just been swabbed with a cotton-wrapped Swiss broach, wet with a very thin solution of the chloro-percha or the oxychloride, as the case may be. When this gold wire is carried to its place it is certain that the root is filled to its extremity as perfectly as it may ever be. The plugger is of soft steel, so that, in a moment, it can be bent at any angle.

In this manner it is possible to fill these canals through a comparatively small tap-hole. This operation requires nice manipulation (there is none in dentistry that requires nicer); but it can be done, and exactly as described here.

If it is not admitted that these minute roots can be accurately filled in this way, I think it must be believed that they can be cleaned and sterilized in the manner described, and that is a clean gain, and, by some, may be considered sufficient; for many believe that if the apical end is closed the roots may safely go unfilled. I do not admit this, but I do believe the thorough cleaning and sterilization of any closed root is more important than its perfect filling.

The objective point in all root-filling is the apical end of the canal. I believe this can be reached with unerring certainty by no other means than by a wire in some form. When this is well filled, the rest of the canal may be poorly filled, and yet the tooth will do well. In general practice I think this end is poorly filled, and the accessible part of the canal is well filled. I am ready to make the assertion that the roots of any tooth freshly devitalized and filled in this way will never give trouble. It is an impossibility if it is accompanied with thorough sterilization. Of course, I would not make any such claim for any tooth that had been dead for some time before the treatment was applied. Dead roots, in which and about which septic influences have been at work, must always remain somewhat uncertain.

You will notice that I have given attention thus far only to very minute canals. There is not time now to say more than a word about the filling of canals of larger size. In fact, there is not very much to say if I had more time. In the early days it was my habit, as it was the habit of most practitioners, I think, to use chloride of zinc, carried to the apex on a shred of cotton, the cotton being left in the root. After hitting upon the idea of gutta-percha points plunged cold into a canal wet with chloro-percha, I used them generally for many years.

For large canals, and those open at the apical ends, I used pointed gold wire dipped in chloro-percha, or barbed and warmed, and wrapped with red gutta-percha, not exactly as described long since by Dr. Morrison, but nearly so.

A few years ago Dr. Howe called the attention of this Society to the fact that gutta-percha is a neutral substance, and, besides having no antiseptic action, is capable of absorption, as shown by the disagreeable odor arising from it when removed from a root-canal and warmed over the flame of a lamp, as well as from its tendency to expand. In fact, this warming is not necessary to demonstrate the fact stated. I recognized the truth of this, and from that time I preceded the gutta-percha points in closed roots with chloride of zinc, plunging the cold, stiff points through this creamy substance, and then filling the root-chamber with it. When these points are surrounded by an envelope of oxychloride of zinc, I think the root remains sterilized, and that this combination makes almost an ideal root-filling. It has the advantage of comparatively easy removal, and that, I think, is an important consideration.

If the canals are only of medium size, and there is danger that the points will be doubled up and not carried through the oxychloride to the apex, then I use gold wire in the manner already described, but wire, of course, of larger size.

If the canals are open at the apex, I use gold wire wrapped with a few shreds of silk and dipped in chloro-percha, and pushed to the apex by exact measurement. If the canals and the opening at the apex are very large, then I use gold wire barbed and wrapped with red gutta-percha, putting it in and taking it out several times to get a sort of impression of the canal, and thus being able to judge a little better of the amount to use, and putting it in finally by exact measurement.

All these gold wires have provision made for their removal, the large one having a notch filed near the end in which an instrument can be engaged, and the little ones being bent double near the end.

Where chloro-percha is used they are all easily removed. They can also be removed where oxychloride is used if they fit the root well, so that only a thin covering of oxychloride exists. But they cannot easily be removed if they are much smaller than the diameter of the canal, so that they are embedded in a thick mass of the oxyphosphite.

Although I make this provision for the removal of these fillings, for it is easily done, yet I never expect to have to do it in any freshly devitalized tooth where I have had the full control of the operation of removing the pulp and filling the root. But with teeth long dead I have no such feeling of security. Such teeth are favorable ones for the use of wax or paraffine, and particularly for a canal paste perfected by Dr. Lord, and used by him for many years. It was used by Dr. Bronson, and since by Dr. Van Woert, and was spoken of very highly by him before this Society. This latter should commend itself to our attention, as it has the great advantage of being easily removed. It is composed of iodol, zinc oxide, carbolated vaseline, and a little oil of cinnamon.

Gold wires are particularly suitable for the successful use of either of these materials.

I cannot close without a further word in reference to oxychloride of zinc. It has been long before the profession, and has probably been more used, skilfully and unskilfully, for root-fillings than any substance known, and it continues to occupy a prominent place to-day as in the past. My own belief is that the good results arising from its use are due mainly to its antiseptic properties. But I consider it a dangerous material to use in roots, the apical ends of which are open. It must be said that it is not altogether easy of removal, and that should lead us to search still further for the more perfect filling. For the large pulp-chamber and as a foundation for the outer filling it is unsurpassed.

